# Evaluation of the BioFire FilmArray Pneumonia Panel Plus to the Conventional Diagnostic Methods in Determining the Microbiological Etiology of Hospital-Acquired Pneumonia

**DOI:** 10.3390/biology11030377

**Published:** 2022-02-27

**Authors:** Noha A. Kamel, Mohammad Y. Alshahrani, Khaled M. Aboshanab, Mervat I. El Borhamy

**Affiliations:** 1 Department of Microbiology, Faculty of Pharmacy, Misr International University (MIU), Cairo P.O. Box 19648, Egypt; noha.ahmed@miuegypt.edu.eg (N.A.K.); mervat.ismail@miuegypt.edu.eg (M.I.E.B.); 2 Department of Clinical Laboratory Sciences, College of Applied Medical Sciences, King Khalid University, P.O. Box 61413, Abha 9088, Saudi Arabia; moyahya@kku.edu.sa; 3 Department of Microbiology and Immunology, Faculty of Pharmacy, Ain Shams University, Organization of African Unity St., Abbassia, Cairo P.O. Box 11566, Egypt; 4 International Medical Center, Clinical Microbiology Laboratory, Cairo P.O. Box 11451, Egypt

**Keywords:** hospital-acquired pneumonia, FilmArray, multiplexed BioFire Pneumonia Panel plus, genetic markers, antibiotic sensitivity

## Abstract

**Simple Summary:**

Hospital-acquired pneumonia (HAP) imposes public health threats because of its high morbidity and mortality rate. Accordingly, this study aimed to evaluate the diagnostic performance of the multiplexed BioFire FilmArray Pneumonia Panel plus (BFPP) for the rapid detection of various clinically relevant respiratory pathogens and genetic markers among 50 patients admitted with HAP to an intensive care unit (ICU) in a tertiary care hospital in Egypt. In comparison to standard culture methods, BFPP showed an overall sensitivity and specificity of 100% and 90%, respectively, with the identification of 11 viral targets (22%) among the tested specimens. The BFPP semi-quantitative analysis showed a concordance rate of 47.4% among positive culture specimens. For the examination of the antibiotic resistance genes, BFPP showed a positive percent agreement (PPA), a negative percent agreement (NPA) and an overall percent agreement (OPA), reaching 97%, 95%, and 95%, respectively, with standard antibiotic sensitivity testing. According to the obtained results, BFPP has the potential to enhance the rapid microbiological diagnosis of HAP cases, tailor appropriate antibiotic therapy, apply antimicrobial stewardship programs, and implement effective infection control measures.

**Abstract:**

Hospital-acquired pneumonia (HAP) is a substantial public health issue that is associated with high mortality rates and is complicated by an arsenal of microbial etiologies, expressing multidrug-resistant phenotypes, rendering relatively limited therapeutic options. BioFire FilmArray Pneumonia Panel plus (BFPP) is a simple multiplexed PCR system that integrates sample preparation, nucleic acid extraction, amplification, and analysis of microbial etiology, with a turnaround time of about one hour. In comparison to standard culture methods, BFPP is simpler, easier to perform, and can simultaneously detect the most common pathogens involved in lower respiratory tract infections (34 targets). Accordingly, we evaluated the diagnostic performance of the multiplexed BFPP for the rapid detection of 27 clinically relevant respiratory pathogens and 7 genetic markers among 50 HAP cases admitted to the intensive care unit (ICU), who submitted mini-bronchoalveolar (mBAL) specimens. In comparison to standard culture methods, BFPP showed an overall sensitivity of 100% [95% CI; 90–100] and overall specificity of 90% [95% CI; 87.4–92.5] among all the tested bacterial targets. BFPP identified 11 viral targets (22%) among the tested specimens. The BFPP semi-quantitative analysis showed a concordance rate of 47.4% among positive culture specimens. For the investigation of the antibiotic resistance genes, BFPP showed a positive percent agreement (PPA), a negative percent agreement (NPA), and an overall percent agreement (OPA), reaching 97% [95% CI; 90–100], 95% [95% CI; 91.5–97], and 95% [95% CI; 93–97], respectively, with standard antibiotic sensitivity testing. In conclusion, BFPP has the potential to enhance the rapid microbiological diagnosis of HAP cases, and could aid in tailoring appropriate antibiotic therapies.

## 1. Introduction

Globally, lower respiratory tract infections (LRTIs), mainly pneumonia cases, are one of the most reported fatal infections among intensive care unit (ICU) patients, and it is estimated that all-cause pneumonia mortality rates might reach 70% or more with the current COVID-19 pandemic [1]. Despite recent progress in the management of such nosocomial infections, hospital-acquired pneumonia (HAP, occurs 48 h or more after hospital admission) and its subcategory—ventilator-associated pneumonia (VAP, occurs 48 h or more after endotracheal intubation)—are still associated with high mortality rates, reaching 20% [2] and 30% or more in surgical ICU [3,4], respectively. In Egypt, VAP occurs among 38.4% of mechanically ventilated patients, with an incidence of 36/1000 ventilator days [5]. In addition to the associated morbidity and high finical burden within hospital settings, the microbiological diagnosis of HAP/VAP remains challenging. Of concern, the difficulty in obtaining a reliable LRT specimen by non-invasive techniques [6], the wide range of microbial profiles (bacteria, virus, or, rarely, fungi) [7], the escalating threats of multidrug-resistant (MDR) bacteria, and the long turnaround time of about 2–3 days to obtain culture results further complicated the ability to diagnose by conventional means [8,9]. However, microbiological methods are necessary in guiding empiric antibiotic regimens and prescribing appropriate treatment [10]. Hence, the development of rapid molecular diagnostic tools, coupled with updated epidemiological profiles of respiratory pathogens, as well as their antimicrobial sensitivity patterns, will be urgently required among HAP patients.

BioFire^®^ FilmArray Pneumonia Panel plus (BFPP, BioMérieux) is a multiplex polymerase chain reaction (PCR) assay that can simultaneously detect 27 clinically relative pneumonia-associated pathogens and 7 antibiotic-resistant targets (1 for extended-spectrum β-lactamases (ESBLs), 5 for carbapenem-resistant Enterobacteriaceae (CRE), and 1 for methicillin-resistant *Staphylococcus aureus* (MRSA)) within one hour. The panel list can identify 15 typical bacteria (4 Gram-positive and 11 Gram-negative), 3 atypical bacteria (*Legionella pneumophila, Mycoplasma pneumoniae,* and *Chlamydia pneumoniae*), and 9 viruses (adenovirus, coronavirus, human metapneumovirus, rhinovirus/enterovirus, influenza A, influenza B, parainfluenza, respiratory syncytial virus (RSV), and Middle East respiratory syndrome coronavirus), with an overall superior sensitivity and specificity over conventional diagnostic methods [11]. The Gram-positive bacteria included in the panel are *Staphylococcus aureus*, *Streptococcus agalactiae*, *Streptococcus pneumoniae,* and *Streptococcus pyogenes*. On the other hand, the Gram-negative bacteria are the *Acinetobacter calcoaceticus–baumannii* complex, *Enterobacter cloacae* complex, *Escherichia coli, Haemophilus influenzae, Klebsiella aerogenes, Klebsiella oxytoca, Klebsiella pneumoniae* group*, Moraxella catarrhalis, Proteus spp., Pseudomonas aeruginosa*, and *Serratia marcescens*. Indeed, the majority of the previously mentioned Gram-negative bacteria, with a special focus on the members of the Enterobacterales, play an important role in urinary tract infections [12,13,14]. Additionally, the panel provides semi-quantitative detection, expressed as genomic copies/mL, for the 15 typical bacteria, to help clinicians quickly determine infection from colonization states and the qualitative detection of other bacteria, as well as respiratory viruses, for better clinical outcomes. Despite of its approval by the Food and Drug Administration (FDA), few data are available on the usefulness of BFPP in quickly determining the complex microbial etiologies of pneumonia infections [15]. In addition, this platform will be useful if urgent infectious targets, such as *Francisella tularensis*, the causative agent of a highly fatal respiratory tularemia, were added to the panel [16]. Therefore, the aim of our study was to evaluate the accuracy of BFPP, compared to the other conventional diagnostic methods, for determining the microbiological etiology of HAP cases admitted to the ICU of a tertiary care hospital in Egypt.

## 2. Materials and Methods

### 2.1. Study Design

A prospective study was conducted from Jan 2021 to June 2021 at the International Medical Center (IMC, Cairo, Egypt), in a tertiary care hospital with 800 beds and 10 medical ICUs (69 beds), to evaluate the characteristic performance of BFPP in determining pneumonia pathogens within hospital settings. The study was conducted in accordance with the ethical principles stated in the Declaration of Helsinki and was approved by the institutional ethics committee, Faculty of Pharmacy, Ain Shams University (ENREC-ASU-2018-72). Admitted patients to all ICUs for 48 h or more, with clinical suspicion of HAP or VAP, were prospectively recruited. Upon clinical diagnosis of nosocomial pneumonia, standard microbiological diagnosis, parallel to BFPP, was performed at a clinical microbiology laboratory to identify causative pathogens and their resistant determinants. To access feasibility of LRTIs, a total of 50 mini-bronchoalveolar lavage (mBAL) specimens were collected through blind insertion of a sterile long suction catheter into the distal airways. Thereafter, 20 mL of isotonic NaCl was introduced into the lung and the aspirate was collected by suction [17]. Laboratory workflow of mini-BAL specimens is delineated in Figure S1.

### 2.2. Standard Microbiological Examination

According to the standard clinical laboratory protocols, mBAL specimens were subjected to Gram staining and culture examination, by streak plate technique, with a calibrated loop on 10^−3^ mL of blood agar, chocolate agar, MacConkey agar, and mannitol salt agar (Becton, Dickinson and Company, Franklin Lakes, NJ, USA). Bacterial isolates that showed growth equal to or above 10^4^ colony forming unit/mL (CFU/mL) were termed as potential pathogens. Before reporting the negative cultures, plates were incubated for an additional 2 days at 35 °C in 5% CO_2_ incubators [18]. In addition to standard biochemical tests, VITEK-2 (BioMérieux, Marcy l’Etoile, Lyon, France) was also used, according to manufacturer’s instructions, to identify bacterial isolates and determine antimicrobial break points. Moreover, PCR technique was performed to confirm the presence of 5 main carbapenemase genes among CRE (*bla*_KPC_, *bla*_IMP_, *bla*_OXA-48_, *bla*_NDM_, and *bla*_VIM_) [19,20] and *bla*_CTX-M_ for ESBLs [21]. Based on a request from healthcare providers, other microbiological tests were performed, whenever needed, to detect other bacterial, viral, and fungal respiratory pathogens.

### 2.3. BFPP Microbiological Examination

In parallel to standard microbiological analysis, BFPP was performed on 50 mBAL specimens, according to the manufacturer’s instructions. Briefly, the pouch was hydrated with the manufacture-supplied hydrated solution, followed by loading of the sample mix (mBAL, in addition to sample buffer), and then running of the pouch. The running process involves lysis of the sample by agitation, extraction/purification by magnetic bead technology, and amplification of the extracted nucleic acid through nested multiplex PCR. For a run to pass, the 2 process controls (RNA process control and quantified standard material control) must both show positive results. The former control was used to check the pouch function and the latter control for semi-quantitative detection of 15 typical bacteria expressed as copies/mL in bin.

### 2.4. Statistical Data Analysis

The correlation between BFPP and standard-of-care tests (culture and antimicrobial sensitivity tests for bacterial pathogens) was investigated in the form of a positive percent agreement (PPA), a negative percent agreement (NPA), and an overall percent agreement (OPA) at 95% confidence intervals (95% CI), by modified Wald method in GraphPad Prism^®^ version 5.00. The PPA = [true positive/(true positive + false negative)] × 100%, NPA = [true negative/(true negative + false positive)] × 100%, and OPA = [(true positive + true negative)/(true positive + false positive + false negative + true negative)] × 100% [22]. If we consider the standard culture method as the reference method, we can use the term sensitivity instead of PPA, and specificity instead of NPA. In the case of LRT infections, it is difficult to depend on standard culture methods alone (other molecular methods are needed to detect viral and unculturable bacteria), so the terms PPA and NPA are more commonly used [22]. For semi-quantitative analysis of the 15 typical bacterial pathogens, we compared culture results (CFU/mL) to BFPP (bin results; copies/mL).

## 3. Results

### 3.1. Evaluation of BFPP and Standard-of-Care Methods to Detect Most Relevant Respiratory Pathogens

A total of 50 mBAL specimens were obtained from 50 patients hospitalized in the ICU for further microbiological investigation of HAP by BFPP and standard-of-care tests. The median age of the patients was 42 years and 54% were male. A summary of the performance data of BFPP and standard microbiological methods to detect the respiratory pathogens is depicted in Table 1. Across the 50 mBAL tested specimens, a total of 44 bacterial targets were detected by both BFPP and standard culture methods. A total of 52 bacterial targets were detected by BFPP alone, and were considered as false positives, while 2 *Pseudomonas putida* (BFPP off-panel) were detected by conventional culture alone. In comparison to standard culture methods, BFPP showed an overall sensitivity of 100% [95% CI; 90–100] and overall specificity of 90% [95% CI; 87.4–92.5]. The PPA/sensitivity for many bacterial targets was 100%, the NPA/specificity ranged from 81 to 98%, and the OPA ranged from 50 to 98%. In this cohort study, five BFPP bacterial targets, i.e., *Klebsiella oxytoca, Streptococcus pyogenes*, *Legionella pneumophila Mycoplasma pneumoniae,* and *Chlamydia pneumoniae*, were not detected by either method.

**Figure 1:**
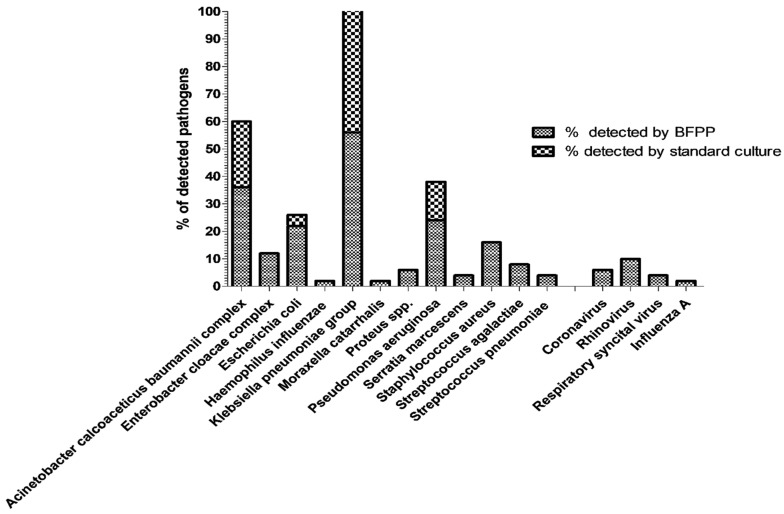
Summary of the percentage of detected pathogens by BioFire Pneumonia Panel plus (BFPP) and standard culture methods.

As shown in Figure 1, the most frequently detected bacteria per specimen, by BFPP and standard culture methods, were *K. pneumoniae* group (56% and 46%), *A. baumannii* (36% and 24%), *P. aeruginosa* (24% and 14%), and *E. coli* (22% and 4%), respectively. The most frequently detected viruses by BFPP were human rhinovirus/enterovirus (10%), followed by coronavirus (6%) and respiratory syncytial virus (4%).

**Figure 2:**
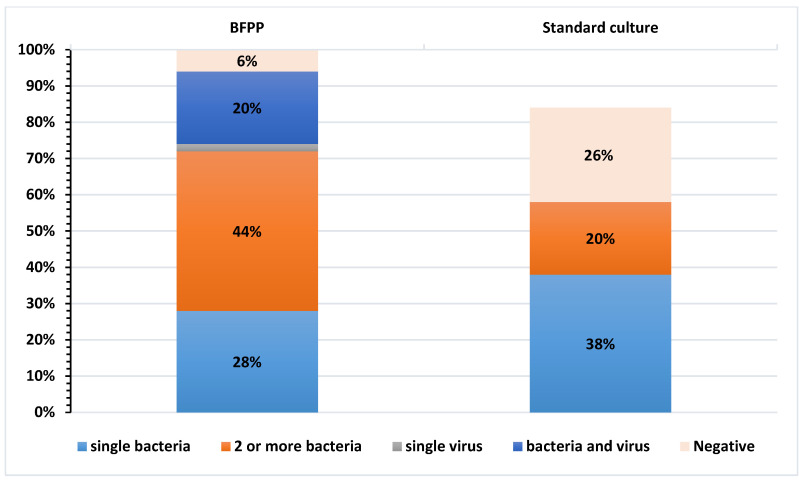
Distribution of respiratory pathogens by BioFire Pneumonia Panel plus and standard culture methods. N.B: viral pathogens were not tested by other standard-of-care methods.

The distribution of single and co-detections of respiratory pathogens, identified by BFPP and culture methods, is depicted in Figure 2. Among the 50 tested specimens, BFPP detected 2 or more bacteria (44%) and single bacteria (28%), and co-detected viral/bacterial infections (20%). In contrast, conventional methods detected single bacteria (38%) and two or more bacteria (20%) among the tested specimens.

### 3.2. Semi-Quantitative Analysis of Bacterial Pathogens by BFPP and Standard Culture Methods

**Figure 3:**
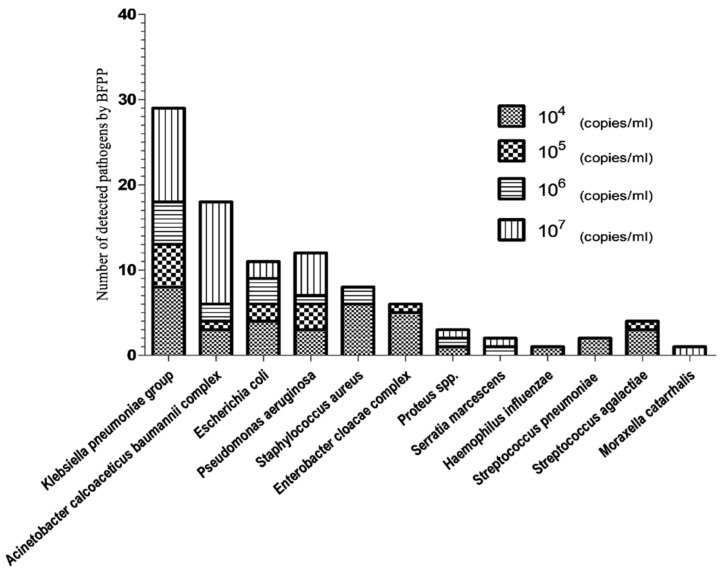
Number of detected pathogens by BFPP.

**Figure 4:**
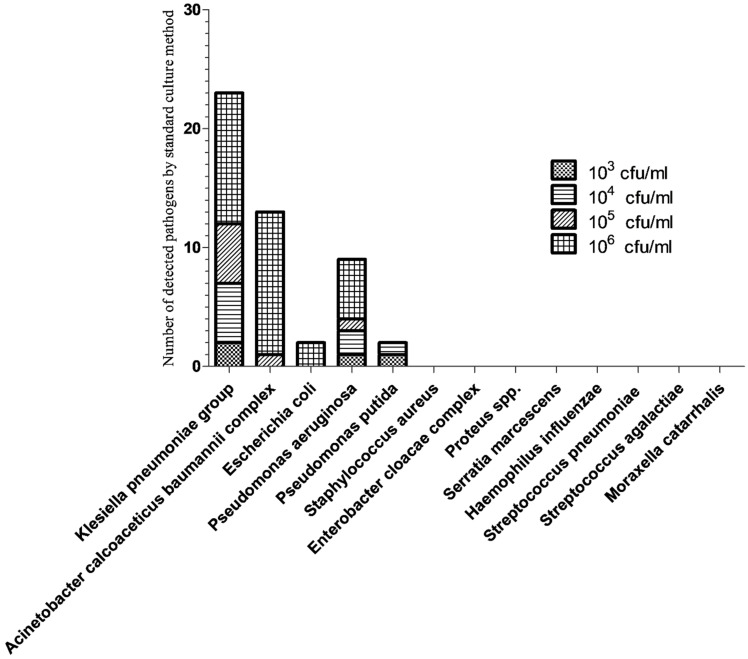
Number of detected pathogens by standard culture method.

The estimates of bacterial amounts, based on BFPP (bin results; copies/mL) and culture methods (CFU/mL), are shown in Figure 3 and Figure 4, respectively. The total number of bacteria detected by BFPP at 10^4^, 10^5^, 10^6^ and 10^7^ copies/mL was 36, 13, 15 and 33, respectively. On the other hand, the total number of bacteria detected by culture methods at 10^3^,10^4^,10^5^ and 10^6^ CFU/mL was 2, 8, 7, and 29, respectively.

### 3.3. Detection of Antimicrobial Resistance Genes

A total of 88 antibiotic resistance genes were detected by BFPP. The most frequently detected were 52 carbapenemase genes (24 NDM; 17 OXA-48; 5 KPC; 5 VIM; 1 IMP), 31 CTX-M, and 5 MecA/C-MREJ. Table 2 compares the accuracy of BFPP to the minimum inhibitory concentration (MIC) provided by VITEK-2. The PPA, NPA, and OPA for the total detected antibiotic resistance genes were 97% [95% CI; 90–100], 95% [95% CI; 91.5–97], and 95% [95% CI; 93–97], respectively.

## 4. Discussion

The diversity of etiological agents associated with HAP has called for adopting rapid molecular diagnostics to allow the prompt identification of respiratory pathogens and improve clinical outcomes. In addition to the qualitative and semi-quantitative detection of a wide range of pneumonia pathogens, BFPP, a nested multiplexed real-time PCR-based method, can rapidly identify the most relevant antimicrobial resistance genes within an hour in hospital settings [23]. In the context of this, we designed a study to compare the performance of BFPP to standard culture methods in the detection of the most common HAP-causing pathogens and their related antibiotic resistance genes.

To overcome the challenges of selecting optimum microbiological diagnostic procedures for HAP patients in the ICU, mBAL, as a simple, low cost method, with relatively minimal disturbance to oxygen levels and hemodynamics during the procedure, was used in our study. Previous studies, comparing the bronchoscope-guided BAL to the less invasive mBAL, had also reported a high concordance rate, ensuring effectiveness of the latter approach to access the distal airway secretions among HAP patients [24,25]. Our data revealed that the overall PPA and NPA for the detection of on-panel bacterial targets by BFPP were 100% and 90%, respectively. Nearly similar results were reported by Edin et al. (PPA = 100% and NPA = 73.2%) [26], Ginocchio et al. (PPA = 93% and NPA = 96%) [27], and Lee et al. (PPA = 90% and NPA = 97.4%) [28]. Although BFPP showed an enhanced overall sensitivity/PPA and specificity/NPA in determining the microbial etiology of HAP patients, the relatively small number of mBAL tested remains one of the major challenges in the current study. On the other hand, based on the nature of our study design (the qualitative detection of microbial etiology among HAP patients), the sample sizes are usually considerably lower in qualitative studies than in quantitative studies. Moreover, a large sample size is a wastage of resources, especially when the new diagnostic test is expensive [29,30]. Lee and co-workers evaluated the performance of BFPP in identifying LRTIs from 51 adult patients in the intensive care unit [28].

The most common pathogens detected by BFPP in our study were *Klebsiella pneumoniae* (28/50), followed by the *Acinetobacter baumannii* complex (18/50) and *Pseudomonas aeruginosa* (12/50). On the other hand, standard culture methods showed low frequency for those pathogens, indicated as 23, 12, and 7 for *Klebsiella pneumoniae*, the *Acinetobacter baumannii* complex, and *Pseudomonas aeruginosa*, respectively. Such pathogen distribution is in line with previous studies that had generally highlighted the role of GNB, mainly *Klebsiella pneumoniae*, as an important cause of HAP, accounting for up to 8% of all hospital-acquired infections. Of concern, the epidemiology and susceptibility patterns of such pathogens are strongly correlated with the studied patient population, and also vary greatly in a geographical and time-dependent manner [12,14]. BFPP uniquely identified certain opportunistic bacterial targets (not detected in our culture results), such as *Staphylococcus aureus* and *Streptococcus pneumonia*, in addition to other GNB that have the potential to cause pneumonia among critically ill patients [31]. Of note, BFPP identified 2 or more bacterial targets (44%) and co-detected bacteria/viruses (20%) among 50 mBAL specimens. Our results were comparable with the incidence of poly-microbial bacterial infections among HAP cases reported by other studies [32,33,34,35].

Out of the 50 tested specimens, 11 viruses were identified by BFPP in the following pattern: rhinovirus/enterovirus (5), coronaviruses (3), RSV (2), and influenza A (1). Such findings must not be under-estimated, as all the detected viral targets may contribute to the appearance of secondary bacterial/fungal co-infections among critically ill patients [36,37]. Of note, the BFPP panel used in our study includes four types of coronaviruses (OC43, NL63, HKU-1, and 229E); however, it still lacks SARS-CoV-2, the current top concern for clinicians and patients. Fortunately, BioFire is working on expanding and updating the respiratory panel to include SARS-CoV-2 and its associated variants.

Another important feature of BFPP is its ability to help clinicians quantify the microbiological load of 15 typical bacterial DNA targets, and, thereby, distinguish clinically significant pneumonia pathogens. However, clinicians must consider that the estimated value of BFPP is about 1 log_10_ higher than the culture results as reported in previous studies. Among all the positive cultures, the concordance rate between culture quantification and BFPP, in this study, reached 47.4%, which is consistent with previous studies that reported 43.6% [38] and 53.6% [28]. This relatively low concordance rate in quantification between BFPP and culture methods could be attributed to the ability of the former method to detect both non-viable and viable organisms, while the latter method is restricted to viable cells only. Notably, many bacterial species, including *Enterobacter cloacae, Staphylococcus aureus*, *Streptococcus agalactiae*, and *Streptococcus pneumoniae*, failed to grow in culture media, and showed low bin values, ranging from 10^4^ to 10^6^ copies/mL. This is attributed to the sensitivity of BFPP to detect low levels of organisms, even if patients had started empirical therapy.

In terms of its accuracy in detecting antimicrobial resistance genes, BFPP showed relatively high agreement when compared to VITEK-2 breakpoints, with PPA [100%, 93%, not applicable], NPA [97%, 85%, 90%], and OPA [98%, 90%, 90%] for carbapenemase producers, ESBLs, and MRSA, respectively. In comparison to other studies [28,39,40,41] that reported on the prevalence of carbapenemase producers and ESBLs among HAP patients, our results revealed a higher rate of detection. This higher resistance rate could be attributed to the large use of antimicrobial agents among ICU patients, leading to more selective pressure and the appearance of different resistant phenotypes. Our study revealed that all carbapenemase producers and ESBL producers (except two isolates) were correctly identified by both BFPP and VITEK-2. Despite this high agreement, we cannot rule out false-negative results among the two ESBL producers, as resistance can be mediated by a variety of enzymes, other than CTX-M, which are still capable of expressing this phenotype. Regarding MRSA detection, exclusively, five isolates harbored the following two DNA targets: methicillin resistance (MecA/C) and staphylococcal cassette chromosome *mec* (SCC*mec*)-*orfX* right-extremity junction (MREJ), which were detected by BFPP. Our findings highlight the sensitivity of BFPP to detect MRSA, even in the absence of *Staphlococcus aureus* by standard culture methods and allow rapid implementation of infection control measures within hospital settings.

In the context of the relatively high resistance rate among GNB, and based on the local antibiogram analysis of our institution, empirical antibiotic therapy, which covers MDR bacteria as ESBL producers and CR, was initially prescribed in the treatment protocols of HAP patients. After comparing BFPP with the standard-of-care results, the physicians treating the patients were responsible for applying antimicrobial stewardship programs through the de-escalation and escalation of antimicrobial agents, whenever indicated. The infection control team also used BFPP as a rapid predictive tool to identify patients who required cohort and isolation.

## 5. Conclusions

BFPP is a pragmatic tool for the rapid etiological diagnosis of nosocomial pneumonia among ICU patients. In comparison to standard culture methods, BFPP showed an overall sensitivity of 100% and specificity of 90% among all the tested bacterial targets. The semi-quantitative detection of 15 bacterial targets and their most relevant antibiotic-resistant genes might have a further positive impact on applying antimicrobial stewardship programs and effective infection control measures.

## Figures and Tables

**Table 1:** Summary on the performance of BFPP and standard microbiological methods to detect the bacterial pathogens.

BFPP Target Organisms (*)		mBAL Specimens [No of BFPP Detections/No of Standard Culture Detections]						
		[+/+]; True Positive	[+/−]; False Positive	[−/+]; False Negative	[−/−]; True Negative	PPA ^a^ %; [95%CI]	NPA ^b^ %; [95%CI]	OPA%; [95%CI]
Bacterial targets	*Acinetobacter calcoaceticus baumannii* complex	12	6	0	32	100%; [72–100]	84.2%; [69–93]	88%; [76–95]
	*Enterobacter cloacae* complex	0	6	0	44	NA	88%; [76–95]	88%; [76–95]
	*Escherichia coli*	2	9	0	39	100%; [29–100]	81%; [68–90]	82%; [69–90]
	*Haemophilus influenzae*	0	1	0	49	NA	98%; [88.5–100]	98%; [88.5–100]
	*Klebsiella pneumoniae* group	23	5	0	22	100%; [83–100]	81.4%; [63–92]	50%; [37–63]
	*Moraxella catarrhalis*	0	1	0	49	NA	98%; [88.5–100]	98%; [88.5–100]
	*Proteus spp.*	0	3	0	47	NA	94%; [83–99]	94%; [83–99]
	*Pseudomonas aeruginosa*	7	5	0	38	100%; [60–100]	88.3%; [75–95.3]	90%; [78.2–96]
	*Serratia marcescens*	0	2	0	48	NA	96%; [86–100]	96%; [86–100]
	*Staphylococcus aureus*	0	8	0	42	NA	84%; [71.2–92]	84%; [71.2–92]
	*Streptococcus agalactiae*	0	4	0	46	NA	92%; [81–97]	92%; [81–97]
	*Streptococcus pneumoniae*	0	2	0	48	NA	96%; [86–100]	96%; [86–100]
	Total bacterial pathogens	44	52	0*	484	100; [90–100]	90%; [87.4–92.5]	91%; [88.4–93.1]

* Two *Pseudomonas putida* were detected by culture only and not detected by BFPP (off-panel). In the case of LRTIs, it is difficult to depend on standard culture methods alone to determine microbial etiology (other molecular methods are needed to detect viral and unculturable bacteria), so terms PPA and NPA are more commonly used. PPA ^a^ and NPA ^b^ should be used in place of sensitivity and specificity, respectively, when the comparator/standard is known to contain uncertainty.

**Table 2:** Correlation between BFPP and VITEK-2 antimicrobial breakpoints for determination of antimicrobial resistance genes.

BFPP Target Organisms Antimicrobial Resistance Genes		mBAL Specimens [No of Results for BFPP/VITEK2 Antimicrobial Breakpoints]						
		[+/+]	[+/−]	[−/+]	[−/−]	PPA %; [95%CI]	NPA%; [95%CI]	OPA%; [95%CI]
Carbapenemase producing Gram negative bacilli ^A^	IMP	0	1	0	49	NA	98%; [88.5–100]	98%; [88.5–100]
	KPC	5	0	0	45	100%; [51–100]	100%; [91–100]	100%; [91.4–100]
	NDM	21	3	0	26	100%; [82–100]	90%; [73–97]	94%; [83–99]
	OXA-48	16	1	0	33	100%; [77–100]	97%; [84–100]	98%; [88.5–100]
	VIM	4	1	0	45	100%; [45–100]	98%; [88–100]	98%; [88.5–100]
	Total carbapenamase producers	46	6	0	198	100%; [91–100]	97%; [94–99]	98%; [95–99]
ESBL producing bacteria ^B^	CTX-M	28	3	2	17	93%; [78–99]	85%; [63–96]	90%; [78.2–96]
Methicillin resistant *Staphylococcus aureus* ^C^	MecA/C and MREJ	0	5	0	45	NA	90%; [78–96]	90%; [78.2–96]
	Total no of detected resistant genes	74	14	2	260	97%; [90–100]	95%; [91.5–97]	95%; [93–97]

^A^ Gram-negative bacilli that showed resistance to any of the tested carbapenems (meropenem, imipenem, and ertapenem) by VITEK-2. ^B^ Gram-negative isolates that showed resistance to any of the tested 3^rd^-generation cephalosporins (ceftriaxone, cefotaxime, and ceftazidime) by VITEK-2. ^C^ Isolates that showed resistance to oxacillin by VITEK-2. KPC, *Klebsiella pneumoniae* carbapenemases; NDM, New Delhi metallo-β-lactamase; IMP, imipenem-resistant Pseudomonas-type carbapenemases; VIM, Verona integron-encoded metallo-β-lactamase; OXA-48, oxacillinase-type carbapenemase; mecA/mecC, a gene A or C that produces a mutated penicillin binding protein coded for methicillin resistance; MREJ, the protein coded by *mec* right-extremity junction (MREJ) (containing the right extremity of SCCmec and orfX, chromosomal *S. aureus* gene).

## Data Availability

Not applicable.

## Supplementary Materials

The following are available online at https://www.mdpi.com/article/10.3390/biology11030377/s1: Figure S1: laboratory workflow of mini-BAL specimens.
