# Supplementary figures and images for: Evaluation of the BioFire FilmArray Pneumonia Panel Plus to the Conventional Diagnostic Methods in Determining the Microbiological Etiology of Hospital-Acquired Pneumonia
Source: Biology (Basel). 2022 Feb 27;11(3):377. doi: 10.3390/biology11030377 (PMC8945136; doi:10.3390/biology11030377)

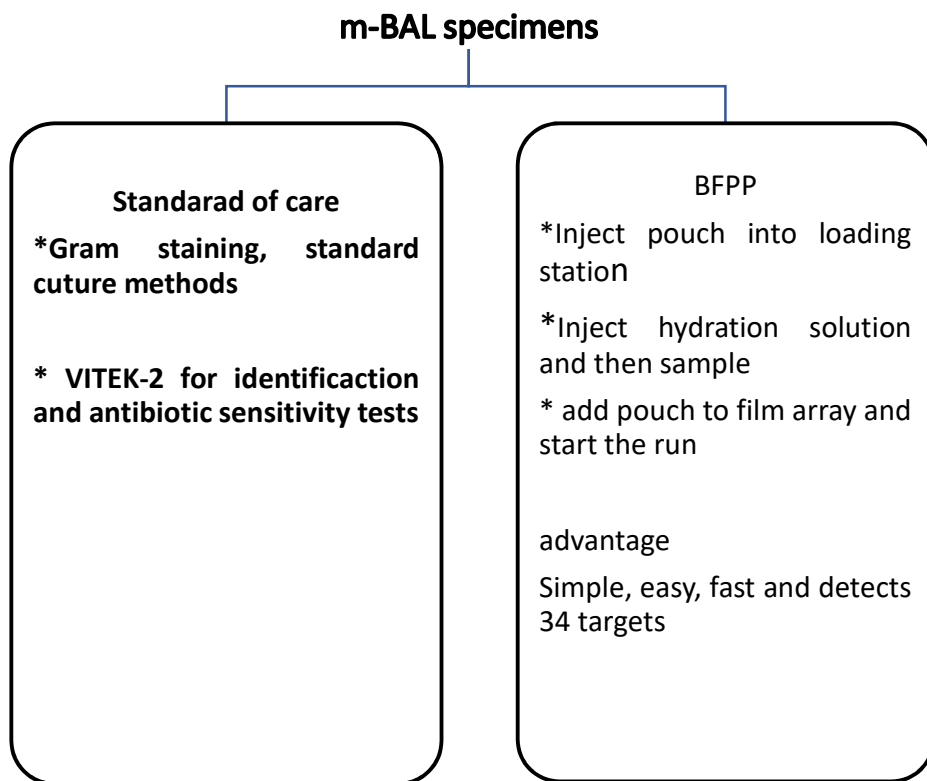

**Figure S1.** Laboratory workflow of mini-BAL specimens

Supplement: Supplementary file 1 [file biology-11-00377-s001.zip › biology-1595002-supplementary.pdf]
